# Retention of Matured Trees to Conserve Fungal Diversity and Edible Sporocarps from Short-Rotation *Pinus radiata* Plantations in Ethiopia

**DOI:** 10.3390/jof7090702

**Published:** 2021-08-27

**Authors:** Tatek Dejene, Emanda Worku, Pablo Martín-Pinto

**Affiliations:** 1 Sustainable Forest Management Research Institute, University of Valladolid, Avda. Madrid 44, 34071 Palencia, Spain; tdejenie@yahoo.com (T.D.); emanda2121@gmail.com (E.W.); 2 Ethiopian Environment and Forest Research Institute (EEFRI), Addis Ababa P.O. Box 30708, Ethiopia; 3 Department of Geography and Environmental Studies, Dilla University, Southern Nations, Nationalities, and Peoples’ Region (SNNPR), Dilla P.O. Box 419, Ethiopia

**Keywords:** edible fungi, conservation, edaphic variables, fungal community, *Pinus radiata*, succession

## Abstract

This study is conducted in the short-rotation plantations from the Afromontane Region of Ethiopia. Sporocarps were sampled weekly in a set of permanent plots (100 m^2^) in young, medium-aged, and mature *Pinus radiata* (Don) plantations. Fungal richness, diversity, and sporocarp yields were estimated. Composite soil samples were also collected from each plot to determine explanatory edaphic variables for taxa composition. We collected 92 fungal taxa, of which 8% were ectomycorrhizal (ECM). Taxa richness, the Shannon diversity index, and ECM species richness were higher in mature stands. Interestingly, 26% of taxa were classified as edible. Sporocarp yield showed increasing trends towards matured stands. OM and C/N ratio significantly affected fungal composition and sporocarp production. The deliberate retention of mature trees in a patch form rather than clear felling of the plantations could be useful to conserve and promote fungal diversity and production, including valuable taxa such as *Morchella*, *Suillus*, and *Tylopilus* in older stands. This approach has important implications for forest floor microhabitats, which are important for macrofungal occurrence and production. Thus, this strategy could improve the economic outputs of these plantations in the Afromontane Region, while the mature trees could serve as a bridge for providing fungal inocula to the new plantations.

## 1. Introduction

In Ethiopia, rapid population growth is stimulating the ever-increasing demand for forest products as well as the expansion of land for crops and grazing, which are among the major causes of the ongoing deforestation in natural forest systems of the country [1,2]. As a result, the creation of plantations of fast-growing trees has become a major forestry practice, thereby reducing pressure on natural forest resources [3,4]. This strategy has led to a rapid expansion in the number of exotic tree species, and more than 1,000,000 ha of land have been planted with exotic species in the past decades [3,5]. Most of these plantations were established as community forests and have considerable potentials for the sustainable production of high-value timber and non-timber forest products (NTFPs) [6], including wild mushrooms [7].

*Pinus radiata* (Don) is the most important non-native commercial timber-producing tree species cultivated in Ethiopia [8]. The species has been established in vast areas covering different agro-climatic zones at elevations ranging from 1500 to 3200 m above sea level (m a.s.l) and mean annual rainfall levels ranging between 700 and 1500 mm, depending on elevation and slope aspect [9]. The majority of *Pinus* plantations have been established in state-owned forests. In terms of the overall plantation area, *Pinus* plantations are the third largest [3,10]. The *P. radiata* tree is preferred, owing to its adaptation to a wide range of ecological conditions and its rapid growth rate, which makes it an important source of round wood for sawn timber, poles, and posts [3,5,10].

In general, *Pinus* species depend on forming symbiotic associations with fungi, which are crucial for their growth and survival [11]. These symbiotic connections favor the uptake of water and nutrients by trees [12,13] and, thus, influence tree productivity [14,15]. Saprotrophic fungi are mainly responsible for the decomposition of organic matter and plant litter in plantation forests of *Pinus* species and, thereby, influence nutrient cycling in these systems [16,17]. In addition to their ecological functions, edible fungi have also become a strategic component in the management of plantation forests. In *Pinus* plantations, edible fungi also represent economically important NTFPs [18] that could provide rural populations with supplementary incomes and generate other benefits [19], particularly for those who depend on forests for their livelihoods.

Although the rotation period for *P. radiata* cultivated in Ethiopia is between 30 and 40 years [20], maximum timber production is achieved when trees are 26–30 years old [3]. Furthermore, plantation management is based on traditional silvicultural systems, with clear felling followed by replanting the preferred management technique [3,21]. Both the short-rotation period and clear-felling may have a direct impact on ecosystem properties and associated fungal communities that are sensitive to this type of management [22]. Furthermore, as forests develop, organic matter production and litter quality change, which results in changes in soil chemical properties [23]. In this sense, stand evolution leads to variations in the woody debris stock, indicating nutrient shedding via litter fall and, consequently, an increase in soil organic matter content in relatively well-developed stands [24]. In general, changes in soil or host tree status may change fungal associations, including their community structures [25,26,27,28]. Thus, macrofungal species observed in plantations could be early-stage fungi, which develop from the spore bank present in the soil before the development of the stand, and late-stage fungi, whose fruiting is enhanced by the new conditions [29]. Previous studies have indicated that clear cutting of plantation trees with long-rotation periods affects the environment and, thus, influences the mycorrhization potential of ectomycorrhizal (ECM) fungi [30]. This could partially explain changes in fungal community composition and fruit body production following clear-felling compared with their pre-harvest status [30]. In addition, the relatively short-rotation period of the plantations and their particular management in Ethiopia might also have an impact on their associated fungal communities. However, as of yet, these impacts are understudied. Thus, identifying mechanisms that control changes in macrofungal communities and production along with the growth of stands through maintaining ecosystem integrity [31,32] while providing wood and non-wood products is imperative to manage Ethiopian plantation forests. In particular, patterns of macrofungal species distribution and changes in their associations over time as stands develop can provide information about the impact of plantation age [25,33], particularly in plantations with short-rotation periods such as those in Ethiopia. This study is the first systematic mycological survey of macrofungal communities inhabiting *P. radiata* stands in Ethiopia. The study was designed to generate information that would be helpful to manage Ethiopian plantation forests that aim to combine the production of timber and NTFPs, especially mushrooms that have both economic and ecological benefits. We hypothesized that fungal taxa composition and sporocarp production would correlate with stand age in a specific way in these short-rotation plantations. In this sense, based on findings in other plantations, independent of the rotation period, we expected that ECM fungal richness in mature *P. radiata* stands would be higher than in younger stands to compensate for nutrient stress [34]. However, we expected that the short-rotation cycle of the *P. radiata* plantations could affect the ECM species composition, particularly of those species dependent on old stands [35]. The shorter rotation period of trees in this study would also result in fertility stress because the nutrient-use efficiency and biomass production of these trees are higher than those of plantation trees with a longer rotation period [36]. Finally, we also expected that the studied *P. radiata* plantations produce valuable edible species, currently underused in Ethiopia, with significant biomass production per sporocarp that could potentially be used as complementary marketable NTFPs by local communities in different part of the country. The overall objective of this study was to characterize the fungal communities present in *P. radiata* stands of three different ages in Menagesha Suba State Forest, central Ethiopia, and to explain their fungal composition based on edaphic variables. Thus, the specific objectives were: (1) to evaluate the macrofungal succession based on richness and diversity variation among stands of three different age classes; (2) to examine sporocarp biomass yield among stands based on edibility and total production; and (3) to identify explanatory edaphic variables that significantly drive the composition of fungal taxa.

## 2. Materials and Methods

### 2.1. Study Area Description

**Figure 1:**
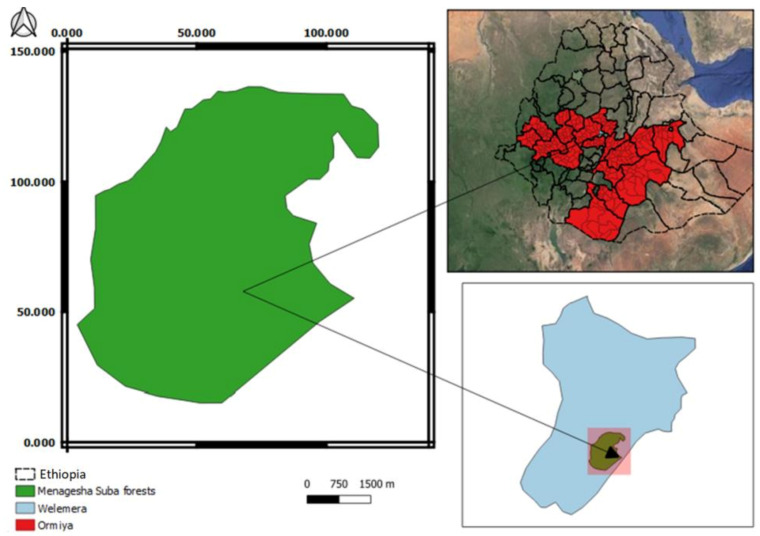
Location map of the study area, Menagesha Suba, central Showa, Oromia National Regional State, Ethiopia.

The study was carried out in Menagesha Suba State Forest in Oromia National Regional State. The forest is located 30 km from Addis Ababa (8°56–9°02′ N and 38°28′–38°36′ E) in the central part of Ethiopia, at 2500–3020 m a.s.l. [37] (Figure 1). The study area is located in the Dega agro-climatic zone, which is characterized by a mean annual rainfall of 1225 mm, which mainly falls from June to September [38]. The mean minimum and maximum monthly temperatures are 6 and 22 °C, respectively [39]. The study area consists of an isolated mountain surrounded by low-lying plains. Soils at higher altitudes are shallow and light brown with a rocky substrate. At lower altitudes, where most of the plantations are located, soils are deep, reddish-brown, and less gravelly [40].

The topography of the area is extremely dissected, with alternating ridges and valleys dominating the landscape. The officially protected area used for forestry covers 9248 ha, of which original natural forests account for 2500 ha and plantation forests of different species account for 1000 ha [38]. Plantations were established on areas that had already been deforested for cultivation or that were affected by unsustainable commercial exploitation, and comprise both indigenous and exotic tree species, including *Juniperus procera* Endl., *Eucalyptus globulus* subsp. *globulus* Labill, *Pinus radiata* (Don), *Pinus patula* Schlechtendal and Chamisso, and *Cupressus lusitanica* Miller [37].

### 2.2. Experimental Design and Sporocarp Sampling

Three different *Pinus radiata* stands were selected based on their ages: 5, 14, and 28 year-old stands, hereafter called young, medium-aged, and mature stands, respectively. Fungal diversity and sporocarp production were determined using transect methods, as described in previous studies [7]. In this study, three 2 × 50 m (100 m^2^) plots were established in each stand, i.e., nine plots in total, as described by Gassibe et al. [41] and Hernández-Rodríguez et al. [29]. Within each of the selected stands, plots were established at least 200 m apart [42]. Plots were laid out randomly in each stand to avoid confounding spatial effects inherent to such a plot-based design [43,44]. Plots were analyzed as independent samples [17].

All sporocarps observed in each plot were harvested weekly during the rainy season in 2020. Fresh weight measurements were carried out in situ. In addition, abundance data (i.e., the number of sporocarps per species) were recorded for each plot. Specimens were photographed in the field, and their ecological characteristics were noted in order to assist and facilitate taxa identification processes. Sample fruit bodies of each species were also taken to the laboratory and dried. Herbaria specimens were used for microscopic taxa identification.

### 2.3. Sporocarp Identification

In the laboratory, the morphological features (tissues and spores) of the fruit bodies were examined with an Optika B-350PL microscope and using appropriate monographs [45,46,47,48,49,50,51,52], to determine the genus and species of the macrofungal specimens. Up-to-date fungal taxa names and authors’ names were obtained from the Mycobank database (http://mycobank.org, accessed on 12 November 2020. Ecological functions at the genus level were assigned using the recent classification compiled by Põlme et al. [53]. In addition, the edibility of the fruiting bodies collected from the study sites was assessed following the criteria used by Bonet et al. [54]. Taxa described in the literature as both non-edible and edible were classified as non-edible. Taxa described in the literature as having doubtful edibility were classified as non-edible. Only species classified as edible by a large majority of the literature consulted were classified as edible fungi.

### 2.4. Soil Sampling and Analysis

To relate macrofungal composition to edaphic variables, soil samples were collected from each of the sample plots established in each of the three stands (Table 1). After clearing and removing plant matter and debris from the soil surface, five soil cores (from the center and the four corners of each plot) were extracted at a depth of 20 cm using an auger. Subsamples collected from each plot were mixed thoroughly, and a composite sample of approximately 500 g was placed in a plastic bag for analysis. Soil samples were dried under a shed until a constant weight was obtained. Dried soil samples were stored in plastic bags at room temperature until they were submitted for laboratory analysis. The analysis was conducted by the Water Works Design and Supervision Enterprises Laboratory Service Sub-Process in the soil fertility section at Addis Ababa, Ethiopia.

The chemical and physical properties of the soil samples were determined using DTPA extraction, KH_2_PO_4_ extraction, Olsen, Kjeldahl digestion, Walkley–Black, ammonium acetate, and instrumental methods. A soil:water (1:2.5) suspension and the supernatant of the same suspension were measured using a pH meter and an electrical conductivity meter, respectively, to determine the soil pH and electrical conductivity (EC). The organic carbon content was determined using wet digestion [55]. The total N content was determined using the Kjeldahl digestion procedure [56].

### 2.5. Statistical Analysis

Shannon’s H’ diversity index, H’ = −∑pi (lnpi) [57], was estimated for each forest, where pi indicates the relative abundance of macrofungal species [58]. Simpson’s diversity, D = 1 − ∑(pi)^2^, where pi is the importance probability in element i, and evenness, J = H’/H’max, where H’ is the number derived from the Shannon diversity index and the H’max is the maximum possible value of H’, were also calculated [59]. In addition, richness values and macrofungal fruit body production levels were also estimated for each stand. All diversity measures were analyzed using the Biodiversity R package [60] in R version 4.0.3 [61].

Differences in soil and sporocarp variables across stands were assessed using linear mixed-effects models (LME) [62], where plot was defined as random and forest age was defined as a fixed factor. LME models were used to prevent false-positive associations due to a relatedness structure in the sampling. A Tukey test was subsequently used to assess significant differences (*p* ≤ 0.05) between stands when needed.

Relationships between macrofungal composition and edaphic variables were visualized using non-metric multidimensional scaling (NMDS) and canonical correspondence analysis (CCA) using CANOCO version 5.0 based on species abundance and soil data matrix. A Monte Carlo permutation test was used to test the statistical significance of environmental variables (999 permutations) in CCA. The isolines of the organic matter (OM), which is referred to by contour lines used to represent the OM gradient on the ordination graphs by connecting points of equal values, provide a good visual representation of the OM content among the three stands. The isolines of the organic matter were calculated using a Loess model, and they were also plotted on the NMDS ordinations using CANOCO version 5.0. The effects of stands on macrofungal composition were analyzed using a permutational multivariate ANOVA (PerMANOVA) based on 999 permutations. A Mantel test, which measures the correlation between group variables, was performed to analyze the effect of stand age and the aggregated edaphic variables. The strength of the difference between stands was measured by R values generated by ANOSIM ranging from 0 to 1, with 1 being the strongest possible difference [63]. The analysis was conducted using PAST software [64]. Effects of edaphic variables on macrofungal community composition were determined based on Bray–Curtis dissimilarity, used to quantify the compositional dissimilarity between *P. radiata* stands, based on counts of taxa at each site. The Bray–Curtis dissimilarity was calculated using the following Formula (1):

BC_ij_ = 1 − [2C_ij_/(S_i_ + S_j_)] (1)

where:

i and j are the two sites,
S_i_ is the total number of species counted on site i,
S_j_ is the total number of species counted on site j,
C_ij_ is the sum of only the lesser counts for each species found in both sites.

The relationship between macrofungal fruit yield and some edaphic variables was determined through linear regression. The analysis used total macrofungal yield as dependent variables while edaphic variables were used as explanatory variables. The power of regression equations was determined by their R-values. A similarity percentages (SIMPER) routine was run to identify the macrofungal species that make the dissimilarity in the community structures [65]. The analysis was performed using PAST software [64].

## 3. Results

### 3.1. Macrofungal Taxa Diversity and Richness

In total, 92 macrofungal taxa were collected, of which, 70 taxa belonged to the Basidiomycota phylum and five to the Ascomycota (Table 2). Of the total taxa collected, 45 (48.91%) were identified up to the species level, 30 (32.61%) up to the genus level, and 17 (18.48%) remained completely unidentified, which might be an indication of both the uniqueness of the forest systems in Ethiopia in terms of diversity of yet undescribed macrofungi species as well as the lack of scientific studies on the local fungal flora in the country. The identified taxa comprised 29 families and 53 genera (Table 2). Family-to-genus and genus-to-species ratios were 0.54 and 0.69, respectively.

The families with the greatest number of species were Agaricaceae (16), Marasmiaceae (7), Polyporaceae (6), Tricholomataceae (6), Geastraceae (4), and Physalacriaceae (4), which together accounted for approximately 57% of the total identified taxa. The distribution of the identified taxa by trophic groups revealed the dominance of saprophytic species, including soil, litter, and wood saprotrophs (86.7%), followed by ECM fungi (8.0%), plant pathogens (2.7%), mycoparasites (1.3%), and moss symbionts (1.3%). According to edibility status, 32% (N = 24) of the total identified taxa collected were edible (Table 2).

**Figure 2:**
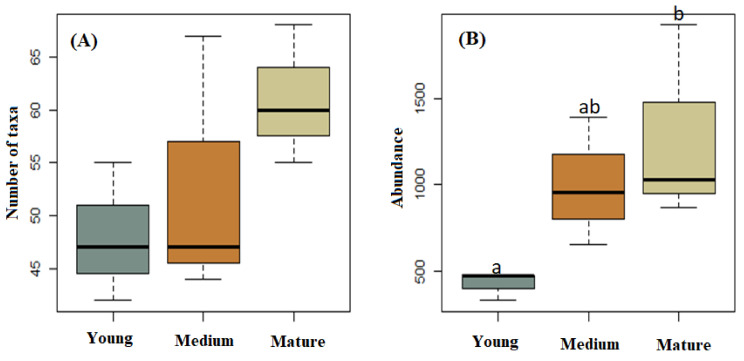
Number of taxa (**A**) and total abundance (**B**) of macrofungal species collected from young, medium-aged, and mature *Pinus radiata* stands in Menagesha Suba, central Showa, Oromia National Regional State, Ethiopia. Boxplot data showing the max and min values. The bar in the box is the standard deviation of the mean. Values with the same letter are not significantly different.

Although there were no significant differences in macrofungal taxa richness among the studied stands (*p* > 0.05), the highest richness values were obtained for plots in the mature stand (taxa = 88) followed by the medium-aged (taxa = 69) and young (taxa = 66) stands (Figure 2A). However, there were significant differences in total macrofungal taxa abundance between the studied stands (F = 7.732, *p* = 0.022). The mean abundance value in the mature stand was significantly higher than that in the young stand (Figure 2B, *p* = 0.022), whereas the abundance in medium-aged stands was not significantly different than that of the young (*p* = 0.059) or mature stands (*p* = 0.711) (Figure 2).

**Figure 3:**
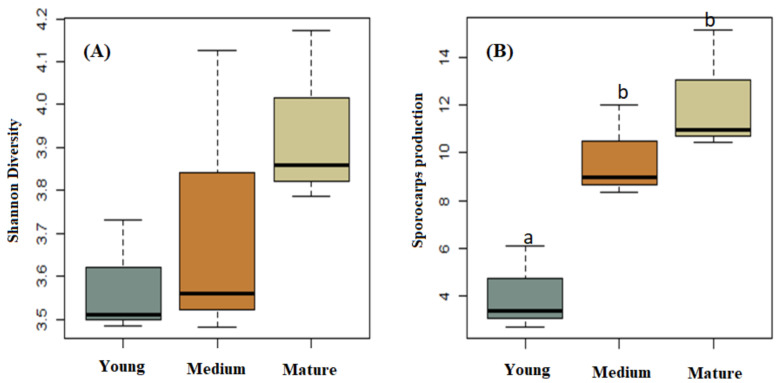
Shannon’s H′ diversity (**A**) and total sporocarp production (kg ha^−1^) (**B**) of macrofungal species collected from young, medium-aged, and mature *Pinus radiata* stands in Menagesha Suba, central Showa, Oromia National Regional State, Ethiopia. Boxplot data showing the max and min values. The bar in the box is the standard deviation of the mean. Values with the same letter are not significantly different.

Shannon’s H′ diversity analysis showed that mature stands had the highest Shannon’s value (4.361); however, there were no significant differences between the three stands (F = 1.646; *p* = 0.269) (Figure 3A). Although only six ECM fungal species were recorded in this study, four of those were only observed in the medium-aged and mature stands (Table 2).

### 3.2. Sporocarp Production

We found significant differences in total sporocarp production among the three stands (F = 13.62; *p* = 0.006; Figure 3B), with significantly greater mean sporocarp production levels in mature (36.58 kg ha^−1^; *p* = 0.006) and medium-aged stands (29.38 kg ha^−1^; *p* = 0.018) than in young stands (12.23 kg ha^−1^).

In total, we collected 25 edible taxa, including ecologically and economically important species such as *Agaricus campestroides*, *Lepista sordida*, *Morchella* sp., *Suillus luteus*, and *Tylopilus niger* (Table 2). The average fresh weight production of edible taxa differed significantly among the three studied stands (F = 6.16; *p* = 0.004), with the highest mean production levels recorded in the mature stand (7.49 kg ha^−1^). This value was significantly higher than that of the young stand (*p*_-mature_ − *p*_-young_ = 0.003) but was not significantly different than that of the medium-aged stand (*p*_-mature_ − *p*_-medium_ = 0.259). Mean sporocarp production was lowest in the young stand (3.29 kg ha^−1^), but this value was not significantly different than that of the medium-aged stands (5.59 kg ha^−1^; *p*_-medium_ − *p*_-young_ = 0.146).

### 3.3. Macrofungal Taxa Composition and Edaphic Variables

**Figure 4:**
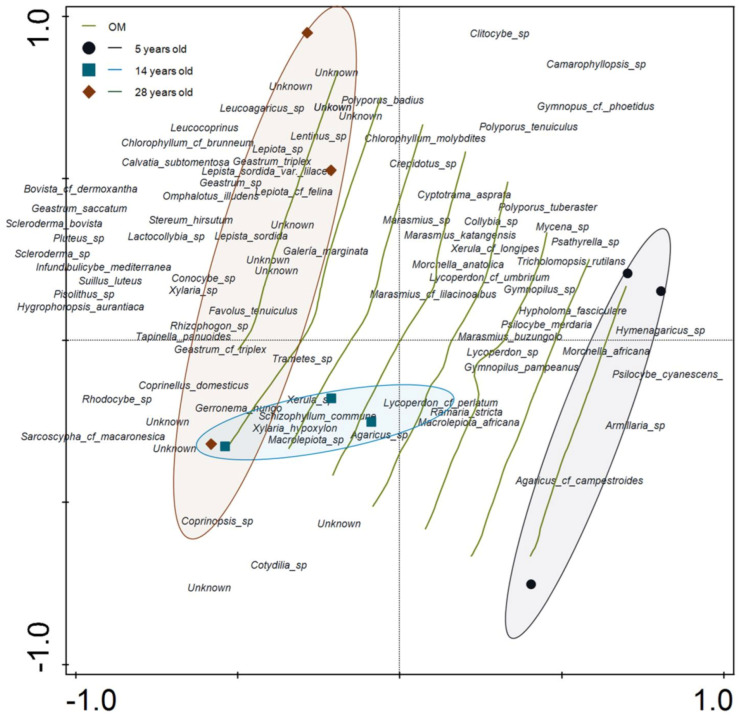
Non-metric multidimensional scaling (NMDS) ordination graph based on dissimilarities calculated using the Bray–Curtis index of macrofungal taxa composition of young (5 years old), medium-aged (14 years old), and mature (28 years old) *Pinus radiata* stands in Menagesha Suba, central Showa, Oromia National Regional State, Ethiopia. The ellipsoids visually cluster stands belonging to the same age group, while the isolines represent the organic matter (OM) values (fitted Loess model R^2^ = 0.86).

NMDS (stress = 0.087) based on Bray–Curtis distance followed by ANOSIM analyses confirmed that the macrofungal taxa composition of the three *P. radiata* stands were significantly different (*p* = 0.004; R = 0.74). However, the analyses showed that the composition of the macrofungal taxa in the young stand was more distinct than those of the medium-aged and mature stands (Figure 4). Pairwise comparisons measuring the strength of the differences in macrofungal composition between the three stands are provided (Table 3).

The Mantel test indicated that macrofungal community structure was significantly affected by stand age (*p* = 0.013; r = 0.49) but was not affected by edaphic variables (*p* = 0.121; r = 0.24). Isolines showing the percentage of soil OM were fitted to the NMDS ordination graph because OM is an important variable that can explain variation in the entire macrofungal taxa composition (Figure 4). Among the soil variables, OM was significantly correlated with taxa composition (r^2^ = 0.68; *p* = 0.03).

**Figure 5:**
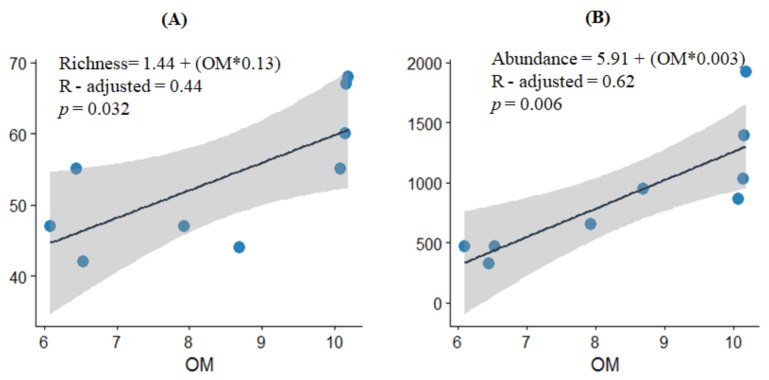
Linear regression models of observed and predicted values of macrofungal richness (**A**) and abundance (**B**) of young, middle aged, and mature *Pinus radiata* stands in Menagesha Suba, central Showa, Oromia National Regional State, Ethiopia. Blue circles represent the observed values of organic matter (OM), black lines indicate the line fit plots, and the shaded areas indicate the 95% confidence intervals.

Additionally, a linear model of the different explanatory variables indicated that the entire macrofungal richness and abundance were significantly predicted by OM (*p* < 0.05; Figure 5). Regressing OM against richness and abundance yielded the equations in Figure 5A and Figure 5B, respectively. In both cases, the model fits the data well and OM explained 44% and 62% of the variation in fungal richness and abundance, respectively. The sign of the coefficient was positive for both, which indicates that as OM increases, richness and abundance also increase. Thus, the average richness and abundance would increase for every 1 unit increase in OM in the studied plantation stands.

**Figure 6:**
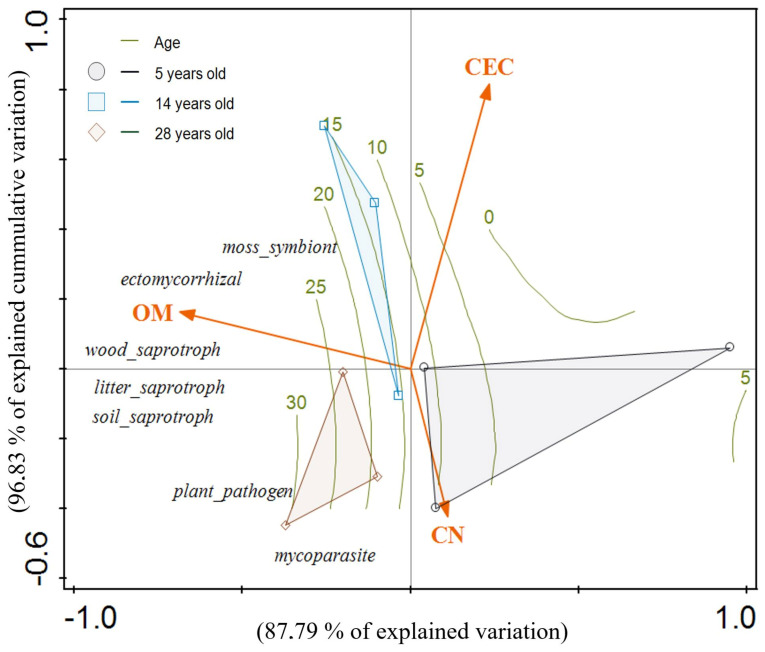
Canonical correspondence analysis ordination plot based on abundance data of functional fungal groups observed in young (5 years old), medium-aged (14 years old), and mature (28 years old) *Pinus radiata* stands in Menagesha Suba, central Showa, Oromia National Regional State, Ethiopia. Plots depicted in the same color are in the same stand. Edaphic variables are shown in orange. The percentage of cumulative explained variation by each axis is shown. Isolines indicate the stand age values (R^2^ = 0.78).

When functional groups were analyzed separately, the soil fertility variables OM, C/N, and CEC were significantly correlated with the macrofungal composition of the *P. radiata* plantations (Table 4). The CCA revealed that both axes explained 96.83% of the cumulative variation in interactions between functional macrofungal composition and soil fertility variables (Figure 6).

The SIMPER analysis also identified macrofungal taxa that make the difference between the three *P. radiata* stands (Table 4). The overall between-group dissimilarity (Bray–Curtis) was 66.62% for the 5 and 15 year-old-stands, 72.53% for the 5 and 28 year-old-stands, and 55.23% for the 14 and 28 year-old-stands. The cumulative contribution of the most influential macrofungal species for the dissimilarity between these forests is shown in Table 5.

## 4. Discussion

This study is the first systematic survey of macrofungal species diversity and production in *P. radiata* plantations in Ethiopia. The study was carried out in forests located in central Ethiopia where the climatic conditions are characterized by high humidity, a variable rainfall pattern, and a prolonged dry season [39]. Patterns of macrofungal species along the age gradients of the forest provide important clues about the underlying mechanisms that structure these communities [66] and are also central for setting conservation priorities for macrofungal species [67] in these forest systems. Although fungi account for a large proportion of global biodiversity [68], reports on macrofungal species from Ethiopian forests are limited. In this study, we collected 92 macrofungal taxa from *P. radiata* plantations in the study area, which is the largest number of fungal taxa reported to date in a plantation of exotic tree species from Ethiopia. For example, Dejene et al. [7] and Dejene et al. [66] recorded 53 and 29 fungal taxa in *P. patula* and *Eucalyptus grandis* plantations, respectively, in the southern part of Ethiopia. Furthermore, Megersa et al. [69] recorded 38 macrofungal taxa under *Cupressus lusitanica* plantations during a three-year survey in the Degaga area, in the south central part of Ethiopia. Thus, our survey findings may indicate that *P. radiata* plantations could play an important ecological role in terms of their fungal diversity, given that fungal richness was much higher than what was observed previously in other non-native tree species plantations in the country. However, taxa identification is still challenging: 18.48% of taxa were unidentified at the genus and species level, indicating that further studies are important to increase our knowledge of the taxonomy and classification of fungi in Ethiopian forests system.

Although our study demonstrated no significant difference in terms of taxa richness and diversity values at different stages of stand development, our data provide valuable baseline information about fungal associations and their succession along age gradients of *P. radiata* plantations in the study area. We observed a trend for increasing taxa richness and diversity values in the more mature stands in agreement with other studies [27,35,70,71]. Previous studies of Ethiopian forest systems have demonstrated that mature *P. patula* and *E. grandis* stands favor more macrofungal species richness and diversity, which might be because as the stands develop, suitable environments are created for macrofungal species [7]. This may lead to the disproportionate fructification of taxa at each successional stage [29] in *P. radiata* stands in the study area. During stand development, each age stage could result in microhabitat variation as the canopy closes [72,73]. Thus, humidity and organic matter accumulation would increase along with stand development, which may enhance the fructification of fungi, which may improve their spatial distribution in terms of taxa richness and diversity, particularly in the older-age classes. This assumption also supports the findings of [74], who noted higher levels of fungal diversity in well-developed stands that had higher levels of canopy closure.

The highest total abundance and sporocarp fresh weight values were obtained from the mature stand, whereas the lowest values were obtained in the young stand, indicating that abundance and the corresponding sporocarp yield increase as the stands mature. These increasing trends can be related to the different availability of substrata with stand age [72], as the forest stand matures, the humus layer develops [73,75,76], and the forest soil increases its capacity to buffer temperature and moisture. Such conditions could enhance fungal growth and fruiting [27]. However, further studies, including observations of the same stands over different years, would be highly desirable to support such trends along stand age gradients, as this would prevent any uncontrolled confounding effect unevenly affecting our results. In this study, we recorded 24 edible taxa, most of which were also found in the mature stand. The relatively high yield of edible taxa might be explained in terms of the abundance and high number of edible taxa observed in the mature plantation. Interestingly, we also recorded important edible species at the study site that have economic significance, such as the morels *Morchella americana* and *M. anatolica*, *S. luteus*, and *T. niger* [18]. In addition, some *Agaricus* species, which have been reported in previous studies [7] and are commonly used by local people in the rural areas of southwest Ethiopia, were also collected in these plantations. Of these important taxa, *S. luteus* was associated with all stand age groups. *S. luteus* is consumed by local people and is also sold in markets at a good commercial price in different developing countries from *Pinus* plantations [77]. For example, in Peru, *S. luteus* is one of the commercial products produced in *Pinus* plantations and is a guarantee of the economic performance of *Pinus* plantations [78] and of the livelihood of local communities [79], thus providing incentives for farmers to plant and manage more trees in their surroundings. Furthermore, *Morchella* spp. produced in Mexico are exported to generate income [77]. Although the overall quantity of sporocarp biomass produced at our study site was low, the yield obtained provides an insight into the potential production levels of sporocarp species, particularly those of potentially marketable species such as *S. luteus*. This also provides a starting point in terms of broadening the management and conservation of plantation forests for the production of NTFPs in Ethiopia. We believe that further studies involving close examination of *P. radiata* stand habitats are needed because there might be still-unknown species with valuable marketable and social potential but unknown uses.

The composition of fungal communities is influenced by different factors, such as forest growth and soil properties, which are key components of forest systems [80,81]. As forests develop, changes occur in the succession of the associated fungal species [82]. Thus, the fungal species could be early-stage, which develop from the spore bank present in the soil before the development of the stand, or late-stage fungi, whose fruiting is enhanced by the new conditions that develop as the forest matures [29]. In this study, we found that the three stands have distinctive fungal communities. Our analyses revealed that the mature and medium-aged *P. radiata* stands have related fungal communities characterized by higher taxa numbers than the young stand. They both had about 25 taxa in common, most of which were characterized as being generalists. The abundance of ECM taxa was higher in these stands, and some of the taxa were exclusively found in medium-aged and mature stands. This finding was probably due to lower soil fertility levels in these stands compared with the young stand, which could increase the dependency of trees on ECM species for water and nutrients [34]. In addition, the positive effect of stand development, which could harmonize high levels of soil humification and a thicker litter layer along with stand development, could be a reason for the presence of generalist fungi in these stands [71,75,76]. For example, *Calvatia* sp., *Geastrum* sp., *Pisolithus* sp., *Pluteus* sp., *Scleroderma* sp., *Suillus* sp., *Tylopilus* sp., and other unknown species were exclusively found in medium-aged and mature stands, indicating that stand age appears to limit the type of taxa, with only those taxa that are able to adapt to the new conditions recorded as the stand develops [83]. Similarly, the young *P. radiata* stand was dominated by fungal species characterized as litter saprotrophs, soil saprotrophs, and wood saprotrophs, such as *Armillaria* sp., *Marasmius* sp., *Polyporus* sp., and *Trametes* sp. However, except for *Armillaria* sp., the other taxa collected from young stands were also found in the medium-aged and mature stands, indicating that most of the taxa collected from the young stand are generalists, and thus, they might also be characteristic of late-stage stands [70].

Due to the short-rotation cycle of the *Pinus* plantation in the study area, knowledge about how the rotation influences the composition of ectomycorrhizal (ECM) species is needed to sustainably manage plantations. In most tropical countries, ECM species form associations exclusively with Pinaceae, coinciding with the natural distribution of these conifers [84]. About 8% of the identified taxa in our study were ECM, and they were abundant in the medium-aged and mature *P. radiata* stands. Gómex-Hernández et al. [85] argue that young stands usually have limited carbohydrate supplies to support mycorrhizal symbionts, which could explain the small number of ECM taxa recorded in the younger stands in this study. Previous studies have suggested that specific ECM taxa can establish relatively early in the early-stage of stand development [86]. Then, as trees in the stand grow, more taxa would dominate the stands, with little difference between mature and older forest age classes [82]. This is true for those stands managed in a long-rotation cycle [35], suggesting that ECM composition can continue to change and may be greater in old stands than in younger stands [82]. However, ECM fungi have different life-history strategies that could be affected by the short-rotation cycle of the plantation. Furthermore, ECM fungi differ in their ability to access nutrients from stands with different levels of growth and complexity [87]. They also differ in their tolerance of different abiotic factors [88], in their tendency to colonize fine roots in different regions of the root system of the plantation [89] and in their ability to disperse and colonize from spores [90]. For these reasons, depending on the age of the plantation, ECM species could be early-, mid- or late-succession species. Studies in other tropical counties indicate that the species found in this study represent early-stage species. For example, *S. luteus* is the most abundant ECM taxa in Argentinean *Pinus* plantations, commonly appearing in stands that are 17–18 years old [84]. Similarly, in Brazil, *S. luteus* fructifications develop in 16–25 year-old *Pinus* stands [91]. Furthermore, Chapela et al. [92] have also reported that *S. luteus* is harvested from *Pinus* plantations in Ecuador when they are 20–30 years old. In all these reports, fungi colonizing plantations of *Pinus* trees that are 16–30 years old are considered to be early-stage colonizers [93]. This suggests that the short-rotation cycle of *Pinus* plantations in Ethiopia could affect the species composition, particularly of those species dependent on old stands [35]. Thus, the retention of mature trees would help conserve macrofungal species that rely on mature trees [30,90,94], particularly ECM species and their mycelial networks. ECM networks have a strong regulating effect on temperate forest ecosystems [95]. Ensuring the persistence of the ECM network in commercially harvested short-rotation plantations has been identified as one of the challenges facing modern forestry [96]. Thus, through tree retention approaches, the occurrence and composition patterns of ECM species across all stages of *Pinus* stand development could be maintained to facilitate their dispersion from nearby intact forests and infection of new roots in younger stands. In addition, there is also the possibility of benefitting from the system by collecting valuable species such as *Suillus*, which could be sold commercially by landowners as well as increasing the economic output of plantations in the study area.

The composition of fungal communities is also influenced by edaphic variables [80,81]. Different fungal taxa are likely to respond to edaphic variables in different ways, depending on their characteristics [97,98], and, thus, in turn, the composition of fungal communities is directly correlated with soil parameters [99]. In this study, organic matter was correlated with the overall macrofungal community from the whole data set. This is likely to be because fungi typically extend their mycelia at the soil–litter interface [100], and thereby, the organic matter influences mycelial outgrowth and network formation [101]. Organic matter also influences the fungal community through its impact on the water-holding capacity of the soil and on nutrient availability [102]. Thus, organic matter may favor more fungal assembly in an area, particularly of saprotrophic fungi. Although data from a single growing season are inadequate to establish any relationship between soil organic matter and macrofungi, our preliminarily findings indicate that the total richness and abundance of macrofungal species is directly related to soil organic matter content, which is consistent with findings reported by Eaton et al. [103].

With regard to macrofungal functional groups, we found that the CEC and the C/N ratio were correlated significantly with composition. Both of these soil properties are on the opposite side of the functional group’s composition, indicating their inverse relationship. This situation is inconsistent with findings reported by Wang and Wang [104]. They showed that a high C/N ratio negatively influenced fungal community structure, probably because a low concentration of N restrains the expansion of fungi. We also found that the CEC is an explanatory factor for macrofungal composition. Although the exact role that the CEC plays in macrofungal composition and sporocarp production is not fully understood, Crabtree et al. [105] observed that fungal species richness was low particularly when the CEC was high. The majority of taxa in our ordinations were directed toward plots with low CEC values. This is probably also because soils with a high CEC are less susceptible to the discharge of base saturation because base saturation is an important factor in the distribution of macrofungal species. Base saturation indicates the proportion of sites occupied by basic cations such as Ca^2+^, Mg^2+^, Na^+^, and K^+^ [106]. These elements are vital in many physicochemical processes, such as photosynthesis [107], and, thus, can affect plant photosynthesis and, hence, the amount of carbon that is available to fungi in the soil [108].

## 5. Conclusions

The *P. radiata* stands analyzed in this study were rich in macrofungal species. Although our data were based on only one year of sampling, we conclude that the richness and diversity of macrofungal taxa were relatively higher in the mature stand than in the other two stands, indicating that fungal diversity values increased with increasing canopy and litter cover. Similarly, the proportion of functional groups was slightly higher in the mature stand of *P. radiata* than in the other stands. We also observed that some species specifically characterized a stand of a particular age, such as *Calvatia* sp., *Geastrum* sp., *Pisolithus* sp., *Pluteus* sp., *Scleroderma* sp., and *Suillus*. There was also a noticeable presence of edible species, which could be potentially marketed in rural areas, providing supplementary incomes to forest managers and local people. Of these species, *Suillus* sp. is reported as edible and growing in abundance in the mature stand. However, stand age and soil parameters were found to affect fungal community composition and, thus, sporocarp production in the study area. Most of the collected ECM species were mainly found in the middle-aged and mature stands of our study areas. This was probably due to the lower soil fertility levels in older stands, which could increase the dependency of trees on mycorrhizal species for water and nutrients, suggesting further studies to justify this claim. Thus, tree management should consider the balance between timber production and fungal community conservation for environmental and production purposes. In this regard, the retention of old trees in clear-cut areas also protects valuable macrofungal species, such as *Suillus* sp. Thus, a tree retention management approach could be used to leave patches of live standing mature trees after the final rotation cut, which could increase habitat availability, such as substratum for saprophytic fungi and increase fungal diversity and edible sporocarp production. The method also provides live standing trees for mycorrhizal species after final rotation cut and thereby provides higher mycorrhizal edible sporocarps production. This could serve as a means of providing the rural population with complementary incomes from the sale of mushrooms, while the mature trees could act as a bridge, providing new plantations with fungal inocula to achieve a balance between timber and fungal production and biodiversity conservation.

## Figures and Tables

**Table 1:** Selected edaphic variables of young, medium-aged, and mature *Pinus radiata* stands in Menagesha Suba, central Showa, Oromia National Regional State, Ethiopia.

Soil Parameter	Stand Age		
	Young	Medium-Aged	Mature
Sand (%)	65.63 ^a^	56.97 ^a^	57.49 ^a^
Silt (%)	19.93 ^a^	15.26 ^a^	16.72 ^a^
Clay (%)	14.44 ^a^	27.78 ^b^	25.79 ^b^
pH-H_2_O	5.33 ^a^	5.54 ^a^	5.62 ^a^
Na (meq/100 g soil)	1.74 ^a^	2.38 ^a^	1.79 ^a^
K (meq/100 g soil)	0.37 ^a^	0.73 ^b^	0.33 ^a^
Ca (meq/100 g soil)	22.62 ^a^	13.70 ^b^	17.81 ^a^
Mg (meq/100 g soil)	6.96 ^a^	5.71 ^b^	5.27 ^b^
CEC (meq/100 g soil)	41.19 ^a^	43.49 ^a^	33.10 ^b^
N (%)	0.37 ^a^	0.52 ^b^	0.32 ^a^
P (mg P_2_O_5_/kg soil)	31.04 ^a^	33.37 ^a^	23.78 ^b^
Organic matter (%)	6.36 ^a^	8.93 ^b^	10.14 ^b^
C/N	10.74 ^a^	10.12 ^a^	15.52 ^b^

Note: Different lowercase letters indicate significantly different soil parameter values among stand ages (*p* < 0.05). CEC: cation exchange capacity, C/N: carbon-to-nitrogen ratio.

**Table 2:** List of fungal taxa collected from *Pinus radiata* stands in Menagesha Suba, central Showa, Oromia National Regional State, Ethiopia.

Species	Phylum	Order	Family	G	E	5y	14y	28y
*Agaricus* cf. *campestroides* Heinem. and Gooss.-Font.	Ba	Agaricales	Agaricaceae	SS	E	x	x	
*Agaricus* sp. Fr.	Ba	Agaricales	Agaricaceae	SS	E		x	
*Armillaria* sp. (Fr.) Staude	Ba	Agaricales	Physalacriaceae	LS	E	x		
*Bovista* cf. *dermoxantha* (Vittad.) De Toni	Ba	Agaricales	Agaricaceae	SS			x	x
*Calvatia subtomentosa* Dissing and M. Lange	Ba	Agaricales	Agaricaceae	SS	E		x	x
*Camarophyllopsis* sp. Herink	Ba	Agaricales	Hygrophoraceae	SS		x		x
*Chlorophyllum* cf. *brunneum* (Farl. and Burt) Vellinga	Ba	Agaricales	Agaricaceae	LS		x	x	x
*Chlorophyllum molybdites* (G. Mey.) Massee	Ba	Agaricales	Agaricaceae	LS		x	x	x
*Clitocybe* sp. (Fr.) Staude	Ba	Agaricales	Tricholomataceae	LS		x		x
*Collybia* sp. (Fr.) Staude	Ba	Agaricales	Tricholomataceae	MP		x		x
*Conocybe* sp. Fayod	Ba	Agaricales	Bolbitiaceae	SS				x
*Coprinellus domesticus* (Bolton) Vilgalys, Hopple and Jacq. Johnson	Ba	Agaricales	Psathyrellaceae	SS		x	x	x
*Coprinopsis* sp. P. Karst.	Ba	Agaricales	Psathyrellaceae	SS		x	x	x
*Cotylidia* sp. Pers.	Ba	Hymenochaetales	Repetobasidiaceae	MS		x	x	x
*Crepidotus* sp. (Fr.) Staude	Ba	Agaricales	Inocybaceae	WS		x	x	x
*Cyptotrama asprata* (Berk.) Redhead and Ginns	Ba	Agaricales	Physalacriaceae	WS		x	x	x
*Favolus tenuiculus* P. Beauv.	Ba	Polyporales	Polyporaceae	WS		x	x	x
*Galerina marginata* (Batsch) Kühner	Ba	Agaricales	Hymenogastraceae	WS		x	x	x
*Geastrum* cf. *triplex* Jungh.	Ba	Geastrales	Geastraceae	LS		x	x	x
*Geastrum saccatum* Fr.	Ba	Geastrales	Geastraceae	LS			x	x
*Geastrum* sp_1_ Pers.	Ba	Geastrales	Geastraceae	LS				x
*Geastrum* sp_2_ Pers.	Ba	Geastrales	Geastraceae	LS		x	x	x
*Gerronema hungo* (Henn.) Degreef and Eyi	Ba	Agaricales	Marasmiaceae	WS		x	x	x
*Gymnopilus pampeanus* (Speg.) Singer	Ba	Agaricales	Strophariaceae	WS	E	x	x	x
*Gymnopilus* sp. P. Karst.	Ba	Agaricales	Strophariaceae	WS		x		x
*Gymnopus foetidus* (Sowerby) J. L. Mata and R. H. Petersen	Ba	Agaricales	Omphalotaceae	LS		x		x
*Hygrophoropsis aurantiaca* (Wulfen) Maire	Ba	Boletales	Hygrophoropsidaceae	LS	E	x	x	x
*Hymenagaricus* sp. Heinem.	Ba	Agaricales	Agaricaceae	SS	E	x	x	x
*Hypholoma fasciculare* (Huds.) P. Kumm.	Ba	Agaricales	Strophariaceae	WS		x	x	x
*Infundibulicybe mediterranea* Vizzini, Contu and Musumeci	Ba	Agaricales	Tricholomataceae	LS		x	x	x
*Lactocollybia* sp. Singer	Ba	Agaricales	Marasmiaceae	LS		x	x	x
*Lentinus* sp. Fr.	Ba	Polyporales	Polyporaceae	WS		x	x	x
*Lepiota* cf. *felina* (Pers.) P. Karst.	Ba	Agaricales	Agaricaceae	LS			x	x
*Lepiota* sp. Singer	Ba	Agaricales	Agaricaceae	LS				x
*Lepista sordida* (Schumach.) Singer	Ba	Agaricales	Tricholomataceae	LS	E			x
*Lepista sordida* var. lilacea (Quél.) Bon	Ba	Agaricales	Tricholomataceae	LS	E		x	x
*Leucoagaricus* sp. Locq. ex Singer	Ba	Agaricales	Agaricaceae	SS	E	x	x	x
*Leucocoprinus* sp. Pat.	Ba	Agaricales	Agaricaceae	SS		x	x	x
*Lycoperdon* cf. *perlatum* Pers.	Ba	Agaricales	Agaricaceae	LS	E	x	x	x
*Lycoperdon* cf. *umbrinum* Pers.	Ba	Agaricales	Agaricaceae	LS	E	x	x	x
*Lycoperdon* sp. Pers.	Ba	Agaricales	Agaricaceae	LS	E	x	x	x
*Macrolepiota africana* (R. Heim) Heinem.	Ba	Agaricales	Agaricaceae	LS	E	x	x	x
*Macrolepiota* sp. Singer	Ba	Agaricales	Agaricaceae	LS	E	x	x	x
*Marasmius buzungolo* Singer	Ba	Agaricales	Marasmiaceae	LS		x	x	x
*Marasmius* cf. *lilacinoalbus* Beeli	Ba	Agaricales	Marasmiaceae	LS		x	x	x
*Marasmius katangensis* Singer	Ba	Agaricales	Marasmiaceae	LS		x	x	x
*Marasmius* sp. Fr.	Ba	Agaricales	Marasmiaceae	LS		x	x	x
*Morchella* cf. *americana* Clowez and C. Matherly	As	Pezizales	Morchellaceae	SS	E	x		x
*Morchella anatolica* Isiloglu, Spooner, Alli and Solak	As	Pezizales	Morchellaceae	SS	E	x		x
*Mycena* sp. (Pers.) Roussel	Ba	Agaricales	Mycenaceae	LS	E	x		x
*Omphalotus illudens* (Schwein.) Bresinsky and Besl	Ba	Agaricales	Marasmiaceae	WS	E	x	x	x
*Pisolithus* sp. (Schwein.) Alb. and Schwein.	Ba	Boletales	Sclerodermataceae	ECM			x	x
*Pluteus* sp. Fr.	Ba	Agaricales	Pluteaceae	LS			x	x
*Polyporus badius* (Pers.) Schwein.	Ba	Polyporales	Polyporaceae	WS	E	x	x	x
*Polyporus tenuiculus* (P. Beauv.) Fr.	Ba	Polyporales	Polyporaceae	WS	E	x	x	x
*Polyporus tuberaster* (Jacq. ex Pers.) Fr.	Ba	Polyporales	Polyporaceae	WS	E	x	x	x
*Psathyrella* sp. Fr. ex Quél.	Ba	Agaricales	Psathyrellaceae	WS		x	x	x
*Psilocybe cyanescens* Wakef.	Ba	Agaricales	Hymenogastraceae	LS		x	x	x
*Psilocybe merdaria* (Fr.) Ricken	Ba	Agaricales	Hymenogastraceae	LS		x	x	x
*Ramaria stricta* (Pers.) Quél.	Ba	Gomphales	Gomphaceae	ECM		x	x	x
*Rhizopogon* sp. Fr. and Nordholm	Ba	Boletales	Rhizopogonaceae	ECM			x	x
*Rhodocybe* sp. Maire	Ba	Agaricales	Entolomataceae	LS			x	x
*Sarcoscypha* cf. *macaronesica* Baral and Korf	As	Pezizales	Sarcoscyphaceae	WS			x	x
*Schizophyllum commune* Fr.	Ba	Agaricales	Schizophyllaceae	WS	E	x	x	x
*Scleroderma* bovista Fr.	Ba	Boletales	Sclerodermataceae	ECM			x	x
*Stereum hirsutum* (Willd.) Pers.	Ba	Russulales	Stereaceae	WS		x	x	x
*Suillus luteus* (L.) Roussel	Ba	Boletales	Suillaceae	ECM	E	x	x	x
*Tapinella panuoides* (Fr.) E.-J. Gilbert	Ba	Boletales	Tapinellaceae	WS		x	x	x
*Trametes* sp. Fr.	Ba	Polyporales	Polyporaceae	WS		x	x	x
*Tricholomopsis rutilans* (Schaeff.) Singer	Ba	Agaricales	Tricholomataceae	WS	E	x	x	x
*Tylopilus niger* (Heinem. and Gooss.-Font.) Wolfe.	Ba	Boletales	Boletaceae	ECM			x	x
*Xerula* cf. *longipes* (Quél.) Maire	Ba	Agaricales	Physalacriaceae	PP		x	x	x
*Xerula* sp. Maire	Ba	Agaricales	Physalacriaceae	PP		x	x	x
*Xylaria hypoxylon* (L.) Grev.	As	Xylariales	Xylariaceae	WS		x	x	x
*Xylaria* sp. Hill ex Schrank	As	Xylariales	Xylariaceae	WS		x	x	x
Undefined sp_1_	Un	Un	Un	Un		x	x	x
Undefined sp_2_	Un	Un	Un	Un		x	x	x
Undefined sp_3_	Un	Un	Un	Un		x	x	x
Undefined sp_4_	Un	Un	Un	Un			x	x
Undefined sp_5_	Un	Un	Un	Un				x
Undefined sp_6_	Un	Un	Un	Un				x
Undefined sp_7_	Un	Un	Un	Un				x
Undefined sp_8_	Un	Un	Un	Un				x
Undefined sp_9_	Un	Un	Un	Un				x
Undefined sp_10_	Un	Un	Un	Un				x
Undefined sp_11_	Un	Un	Un	Un			x	x
Undefined sp_12_	Un	Un	Un	Un		x	x	x
Undefined sp_13_	Un	Un	Un	Un		x		x
Undefined sp_14_	Un	Un	Un	Un		x		x
Undefined sp_15_	Un	Un	Un	Un		x		x
Undefined sp_16_	Un	Un	Un	Un		x		x
Undefined sp_17_	Un	Un	Un	Un		x	x	x

Note: 5y: 5 year-old stands; 14y: 14 year-old stands; 28y: 28 year-old stands; ECM: ectomycorrhizae; SS: soil saprotroph; LS: litter saprotroph; WS: wood saprotroph, PP: plant pathogen; MP: mycoparasite; MS: moss symbionts; E: edible; G: guild; Ba: Basidiomycota; As: Ascomycota; Un: undefined.

**Table 3:** ANOSIM pairwise comparisons of macrofungal composition between young, medium-aged, and mature *Pinus radiata* stands based on Bray–Curtis distance measures (global R-value = 0.74; *p* = 0.004).

Pairwise Comparisons	R Values	*p*
Young and medium-aged stands	0.889	0.001
Young and mature stands	0.815	0.001
Medium-aged and mature stands	0.519	0.009

**Table 4:** Significance of explanatory variables for functional macrofungal compositions based on macrofungal abundance data. Highly significant effects are shown in bold (*p* < 0.05).

Variables	Explains %	Contribution %	Pseudo-F	*p*
OM	30.4	30.4	3.1	0.01
C/N	19.5	19.5	2.3	0.08
CEC	20.6	20.6	3.5	0.03

**Table 5:** Summary of similarity percentage (SIMPER) results based on Bray–Curtis measures showing the cumulative total contribution (30% cut-off) and the contribution (%) of the most influential species to the dissimilarity between the young, middle-aged, and mature *Pinus radiata* stands in Menagesha Suba, central Showa, Oromia National Regional State, Ethiopia.

Species	Individual Contribution to the Dissimilarity	Cumulative Contribution to the Dissimilarity	Edibility Status
**Young and Medium-Aged Stands**			
*Infundibulicybe mediterranea*	4.19	4.18	
*Polyporus tuberaster*	2.83	7.01	E
*Ramaria stricta*	2.79	9.81	
*Tapinella panuoides*	2.72	12.52	
*Lactocollybia* sp.	2.63	15.15	
*Sarcoscypha* cf. *macaronesica*	2.56	17.71	
*Hygrophoropsis aurantiaca*	2.42	20.13	E
*Undefined_1_*	2.11	22.24	
*Hymenagaricus* sp.	1.94	24.18	E
Undefined_3_	1.94	26.12	
*Stereum hirsutum*	1.89	28.01	
Undefined_13_	1.86	29.87	
**Young and Mature Stands**			
*Psilocybe cyanescens*	2.39	2.39	
*Pluteus* sp.	2.38	4.77	
*Infundibulicybe mediterranea*	2.34	7.11	
*Conocybe* sp.	2.28	9.39	
*Geastrum triplex*	2.27	11.67	
*Omphalotus illudens*	2.18	13.85	E
*Lactocollybia* sp.	2.13	15.98	
*Scleroderma bovista*	2.04	18.03	
*Ramaria stricta*	1.86	19.88	
*Gymnopus foetidus*	1.72	21.60	
*Schizophyllum commune*	1.71	23.31	E
*Tylopilus niger*	1.69	25.00	E
*Chlorophyllum* cf. *brunneum*	1.67	26.67	
*Geastrum saccatum*	1.58	28.25	
Undefined_2_	1.58	29.83	
**Medium-Aged and Mature Stands**			
*Infundibulicybe mediterranea*	3.18	3.18	
*Ramaria stricta*	2.34	5.51	
*Geastrum triplex*	2.22	7.73	
*Lactocollybia* sp.	2.20	9.94	
*Conocybe* sp.	2.13	12.07	
*Polyporus tuberaster*	1.97	14.03	E
*Psilocybe cyanescens*	1.96	15.99	
*Sarcoscypha* cf. *macaronesica*	1.62	17.61	
*Schizophyllum commune*	1.61	19.22	E
Undefined_1_	1.59	20.81	
*Marasmius* sp.	1.51	22.32	
*Tapinella panuoides*	1.48	23.80	
*Gymnopus foetidus*	1.43	25.23	
Undefined_17_	1.42	26.66	
*Pluteus* sp.	1.42	28.08	
*Marasmius* cf. *lilacinoalbus*	1.40	29.48	

## Data Availability

Not applicable.

## References

[1] Badege B. (2001). Deforestation and land degradation in the Ethiopian highlands: A strategy for physical recovery. Northeast Afr. Stud..

[2] Jaleta D., Mbilinyi B., Mahoo H., Lemenih M. (2016). Eucalyptus expansion as relieving and provocative tree in Ethiopia. J. Agric. Ecol. Res. Int..

[3] Bekele M. (2011). Forest Plantations and Woodlots in Ethiopia.

[4] Zewdie M., Olsson M., Verwijst T. (2009). Above-ground biomass production and allometric relations of *Eucalyptus globulus* Labill. coppice plantations along a chronosequence in the central highlands of Ethiopia. Biomass Bioenergy.

[5] Tesfaye M.A., Gardi O., Anbessa T.B., Blaser J. (2020). Aboveground biomass, growth and yield for some selected introduced tree species, namely *Cupressus lusitanica*, *Eucalyptus saligna* and *Pinus patula* in Central Highlands of Ethiopia. J. Ecol. Environ..

[6] Bekele M., Lemenih M. (2008). Participatory Forest Management Best Practices, Lesson Learnt and Participatory Forest Management Best Practices, Lesson Learnt and challenges encountered: The Ethiopian and Tanzanian Experiences.

[7] Dejene T., Oria-de-Rueda J.A., Martín-Pinto P. (2017). Fungal diversity and succession following stand development in Pinus patula Schiede ex Schltdl. & Cham. plantations in Ethiopia. For. Ecol. Manag..

[8] Hvidberg-Hansen H. (1978). The growth of some exotic forest trees in the Munessa forest, Ethiopia. Commonw. For. Assoc..

[9] Mesfin D., Sterba H. (1996). A yield table model for the growth of *pinus patula* in ethiopia. J. Trop. For. Sci..

[10] Gezahgne A. (2003). Diseases of Exotic Plantation Forestry Trees in Ethiopia. Ph.D. Thesis.

[11] Halling R.E. (2001). Ectomycorrhizae: Co-Evolution, Significance, and biogeography. Ann. Missouri Bot. Gard..

[12] Egli S. (2011). Mycorrhizal mushroom diversity and productivity—An indicator of forest health?. Ann. For. Sci..

[13] Hall I.R., Yun W., Amicucci A. (2003). Cultivation of edible ectomycorrhizal mushrooms. Trends Biotechnol..

[14] Deacon J. (2006). Fungal Biology.

[15] Westover K.M., Bever J.D. (2001). Mechanisms of plant species coexistence: Roles of rhizosphere bacteria and root fungal pathogens. Ecology.

[16] Deacon J. (2009). Fungal Biology.

[17] Ruiz-Almenara C., Gándara E., Gómez-Hernández M. (2019). Comparison of diversity and composition of macrofungal species between intensive mushroom harvesting and non-harvesting areas in Oaxaca, Mexico. PeerJ.

[18] Boa E. (2004). Wild Edible fungi: A Global Overview of Their Use and Importance to People.

[19] Oria-De-Rueda J.A., Martín-Pinto P., Olaizola J. (2008). Bolete Productivity of cistaceous scrublands in Northwestern Spain. Econ. Bot..

[20] Tesfaye M.A., Bravo-Oviedo A., Bravo F., Kidane B., Bekele K., Sertse D. (2015). Selection of tree species and soil management for simultaneous fuelwood production and soil rehabilitation in the Ethiopian central highlands. Land Degrad. Dev..

[21] Mekonnen Z., Kassa H., Lemenh M., Campbell B. (2007). The role and management of Eucalyptus in Lode Hetosa district, Central Ethiopia. For. Trees Livelihoods.

[22] Paz C.P., Gallon M., Putzke J., Ganade G. (2015). Changes in macrofungal communities following forest conversion into tree plantations in Southern Brazil. Biotropica.

[23] Lei J., Du H., Duan A., Zhang J. (2019). Effect of stand density and soil layer on soil nutrients of a 37-year-old *Cunninghamia lanceolata* plantation in Naxi, Sichuan Province, China. Sustainability.

[24] Soong J.L., Janssens I.A., Grau O., Margalef O., Stahl C., Van Langenhove L., Urbina I., Chave J., Dourdain A., Ferry B. (2020). Soil properties explain tree growth and mortality, but not biomass, across phosphorus-depleted tropical forests. Sci. Rep..

[25] Ágreda T., Cisneros Ó., Águeda B., Fernández-Toirán L.M. (2014). Age class influence on the yield of edible fungi in a managed Mediterranean forest. Mycorrhiza.

[26] Castaño C., Lindahl B.D., Alday J.G., Hagenbo A., Martínez de Aragón J., Parladé J., Pera J., Bonet J.A. (2018). Soil microclimate changes affect soil fungal communities in a Mediterranean pine forest. New Phytol..

[27] Fernández-Toirán L., Agreda T., Olano J. (2006). Stand age and sampling year effect on the fungal fruit body community in *Pinus pinaster* forests in central Spain. Can. J. Bot..

[28] Dymov A.A., Startsev V.V., Milanovsky E.Y., Valdes-Korovkin I.A., Farkhodov Y.R., Yudina A.V., Donnerhack O., Guggenberger G. (2021). Soils and soil organic matter transformations during the two years after a low-intensity surface fire (Subpolar Ural, Russia). Geoderma.

[29] Hernández-Rodríguez M., Oria-de-Rueda J.A., Martín-Pinto P. (2013). Post-Fire fungal succession in a Mediterranean ecosystem dominated by *Cistus ladanifer* L.. For. Ecol. Manag..

[30] Durall D.M., Gamiet S., Simard S.W., Kudrna L., Sakakibara S.M. (2006). Effects of clearcut logging and tree species composition on the diversity and community composition of epigeous fruit bodies formed by ectomycorrhizal fungi. Can. J. Bot..

[31] Lindenmayer D.B., Franklin J.F., Lõhmus A., Baker S.C., Bauhus J., Beese W., Brodie A., Kiehl B., Kouki J., Pastur G.M. (2012). A major shift to the retention approach for forestry can help resolve some global forest sustainability issues. Conserv. Lett..

[32] Nyland R.D. (2002). Silviculture: Concepts and Applications.

[33] Trudell S.A., Edmonds R.L. (2004). Macrofungus communities correlate with moisture and nitrogen abundance in two old-growth conifer forests, Olympic National Park, Washington, USA. Can. J. Bot..

[34] Castaño C., Dejene T., Mediavilla O., Geml J., Andres J., Oria-de-Rueda J., Martín-Pinto P. (2019). Changes in fungal diversity and composition along a chronosequence of *Eucalyptus grandis* plantations in Ethiopia. Fungal Ecol..

[35] Kranabetter J.M., Friesen J., Gamiet S., Kroeger P. (2005). Ectomycorrhizal mushroom distribution by stand age in western hemlock—Lodgepole pine forests of northwestern British Columbia. Can. J. For. Res..

[36] Kimaro A.A., Timmer V.R., Mugasha A.G., Chamshama S.A.O., Kimaro D.A. (2007). Nutrient use efficiency and biomass production of tree species for rotational woodlot systems in semi-arid Morogoro, Tanzania. Agrofor. Syst..

[37] Senbeta F., Teketay D. (2001). Regeneration of indigenous woody species under the canopies of tree plantations in Central Ethiopia. Trop. Ecol..

[38] Zewdie A. (2007). Comparative Floristic Study on Menagesha Suba State Forest on Years 1980 and 2006. Master’s Thesis.

[39] Duguma L.A., Hager H., Gruber M. (2009). The community-state forest interaction in Menagesha Suba Area, Ethiopia: The challenges and possible solutions. For. Trees Livelihoods.

[40] Bekele T. (1994). Vegetation Ecology of Remnant Afromontane Forests on the Central Plateau of Shewa, Ethiopia.

[41] Gassibe P.V., Fabero R.F., Hernández-Rodríguez M., Oria-de-Rueda J.A., Martín-Pinto P. (2011). Fungal community succession following wildfire in a Mediterranean vegetation type dominated by *Pinus pinaster* in Northwest Spain. For. Ecol. Manag..

[42] Luoma D.L., Frenkel R.E., Trappe J.M. (1991). Fruiting of hypogeous fungi in Oregon Douglas-Fir forests: Seasonal and habitat variation. Mycologia.

[43] Hiiesalu I., Bahram M., Tedersoo L. (2017). Plant species richness and productivity determine the diversity of soil fungal guilds in temperate coniferous forest and bog habitats. Mol. Ecol..

[44] Rudolph S., Maciá-Vicente J.G., Lotz-Winter H., Schleuning M., Piepenbring M. (2018). Temporal variation of fungal diversity in a mosaic landscape in Germany. Stud. Mycol..

[45] Antonin V. (2007). Fungus Flora of Tropical Africa, Volume 1: Monograph of Marasmius, Gloiocephala, Palaeocephala and Setulipes in Tropical Africa.

[46] Hama O., Maes E., Guissou M.I., Ibrahim D.M., Barrage M., Parra L.A., Raspe O., De Kesel A. (2010). *Agaricus subsaharianus*, une nouvelle espèce comestible et consommée au Niger, au Burkina Faso et en Tanzanie. Cryptogamie Mycologie.

[47] Heinemann P. (1956). Flore Iconographique des Champignons du Congo, Fasc. 5: Agaricus 1.

[48] Hjortstam K., Ryvarden L. (1996). New and interesting wood-inhabiting fungi (*Basidiomycotina-Aphyllophorales*) from Ethiopia. Mycotaxon.

[49] Morris B. (1990). An annotated check-list of the macrofungi of Malawi. Kirkia.

[50] Pegler D. (1969). Studies on African Agaricales: II. Kew Bull..

[51] Rammeloo J., Walley R. (1993). The edible fungi of Africa South of the Sahara: A literature Survey. Econ. Bot..

[52] Singer R. (1965). Marasmius. Flore Inconographique des Champignons du Congo.

[53] Põlme S., Abarenkov K., Henrik Nilsson R., Lindahl B.D., Clemmensen K.E., Kauserud H., Nguyen N., Kjøller R., Bates S.T., Baldrian P. (2020). Fungal traits: A user-friendly traits database of fungi and fungus-like stramenopiles. Fungal Divers..

[54] Bonet J.A., Fischer C.R., Colinas C. (2004). The relationship between forest age and aspect on the production of sporocarps of ectomycorrhizal fungi in *Pinus sylvestris* forests of the central Pyrenees. For. Ecol. Manag..

[55] Walkley A., Black I.A. (1934). An examination of the digestion method for determining soil organic matter and a proposed modification of the chromic acid titration method. Soil Sci..

[56] Kim H.T. (1996). Soil Sampling, Preparation and Analysis.

[57] Shannon C.E., Weaver W. (1949). The Mathematical Theory of Communication.

[58] Kent M., Coker P. (1993). Vegetation Description and Analysis: A Practical Approach.

[59] Magurran A.E. (1988). Ecological Diversity and its Measurement.

[60] Kindt R., Coe R. (2005). Tree Diversity Analysis. A Manual and Software for Common Statistical Methods for Ecological and Biodiversity Studies.

[61] (2020). R Core Team A Language and Environment for Statistical Computing.

[62] Pinheiro J., Bates D., DebRoy S., Sarkar D., R Core Team (2016). Nlme: Linear and Nonlinear Mixed Effects Models. R Package Version 3.1-128. https://cran.r-project.org/web/packages/nlme/index.html.

[63] Clarke K.R., Gorley R.N., Somerfield P.J., Warwick R.M. (2014). Change in Marine Communities: An Approach to Statistical Analysis and Interpretation.

[64] Hammer Ø., Harper D.A.T., Ryan P.D. (2001). PAST: Paleontological statistics software package for education and data analysis. Palaeontol. Electron..

[65] Parravicini V., Micheli F., Montefalcone M., Villa E., Morri C., Bianchi C.N. (2010). Rapid assessment of epibenthic communities: A comparison between two visual sampling techniques. J. Exp. Mar. Bio. Ecol..

[66] Dejene T., Oria-de-Rueda J.A., Martín-Pinto P. (2017). Fungal diversity and succession under *Eucalyptus grandis* plantations in Ethiopia. For. Ecol. Manag..

[67] Mueller G.M., Schmit J.P. (2007). Fungal biodiversity: What do we know? what can we predict?. Biodivers. Conserv..

[68] Green J., Bohannan B.J.M. (2006). Spatial scaling of microbial biodiversity. Trends Ecol. Evol..

[69] Megersa S., Gure A., Feleke S., Alemu M. (2017). Macrofungi species richness and diversity in Dagaga and Gambo plantation and natural forests of Arsi Forest Enterprise, Oromia, Ethiopia. Imp. J. Interdiscip. Res..

[70] Gassibe P.V., Oria-de-Rueda J.A., Martín-Pinto P.P. (2015). pinaster under extreme ecological conditions provides high fungal production and diversity. For. Ecol. Manag..

[71] Mediavilla O., Oria-de-Rueda J.A., Martín-Pinto P. (2014). Changes in sporocarp production and vegetation following wildfire in a Mediterranean Forest Ecosystem dominated by *Pinus nigra* in Northern Spain. For. Ecol. Manag..

[72] Oria-de-Rueda J.A., Hernández-Rodríguez M., Martín-Pinto P., Pando V., Olaizola J. (2010). Could artificial reforestations provide as much production and diversity of fungal species as natural forest stands in marginal Mediterranean areas?. For. Ecol. Manag..

[73] Dove N.C., Keeton W.S. (2015). Structural complexity enhancement increases fungal species richness in northern hardwood forests. Fungal Ecol..

[74] Sysouphanthong P., Thongkantha S., Zhao R., Soytong K., Hyde K.D. (2010). Mushroom diversity in sustainable shade tea forest and the effect of fire damage. Biodivers. Conserv..

[75] Pinna S., Gévry M.F., Côté M., Sirois L. (2010). Factors influencing fructification phenology of edible mushrooms in a boreal mixed forest of Eastern Canada. For. Ecol. Manag..

[76] Toivanen T., Markkanen A., Kotiaho J.S., Halme P. (2012). The effect of forest fuel harvesting on the fungal diversity of clear-cuts. Biomass Bioenergy.

[77] Pérez-Moreno J., Martínez-Reyes M., Yescas-Pérez A., Delgado-Alvarado A., Xoconostle-Cázares B. (2008). Wild mushroom markets in central Mexico and a case study at Ozumba. Econ. Bot..

[78] Peter T., Maria H., Albino Q., Amarilda L. (2012). Native Mushrooms, Local Knowledge, and Potential for Food and Health in the Peruvian Andes.

[79] Elizabeth M.-E., Felipe R.-S., Maribel I.-M. (2018). Conocimiento popular acerca de la K’allampa de pino (*Suillus luteus* (L.) Roussel) en la localidad de Alalay, Mizque (Cochabamba, Bolivia): Un ejemplo de diálogo de saberes. Asociacion Etnobiologica Mexicana.

[80] Liang Y., He X., Chen C., Feng S., Liu L., Chen X., Zhao Z., Su Y. (2015). Influence of plant communities and soil properties during natural vegetation restoration on arbuscular mycorrhizal fungal communities in a karst region. Ecol. Eng..

[81] Rillig M., Aguilar-Trigueros C., Joana B., Erik V., Veresoglou S., Anika L. (2015). Plant root and mycorrhizal fungal traits for understanding soil aggregation. New Phytol..

[82] Smith J.E., Molina R., Huso M.M., Luoma D.L., McKay D., Castellano M.A., Lebel T., Valachovic Y. (2002). Species richness, abundance, and composition of hypogeous and epigeous ectomycorrhizal fungal sporocarps in young, rotation-age, and old-growth stands of Douglas-fir (*Pseudotsuga menziesii*) in the Cascade Range of Oregon, U.S.A. Can. J. Bot..

[83] Greeshma A.A., Sridhar K.R., Pavithra M., Ghate S.D. (2016). Impact of fire on the macrofungal diversity in scrub jungles of south-west India. Mycology.

[84] Barroetaveña C., La Manna L., Alonso M.V. (2008). Variables affecting *Suillus luteus* fructification in ponderosa pine plantations of Patagonia (Argentina). For. Ecol. Manag..

[85] Gómez-Hernández M., Williams-Linera G. (2011). Diversity of macromycetes determined by tree species, vegetation structure, and microenvironment in tropical cloud forests in Veracruz, Mexico. Botany.

[86] Dighton J., Poskitt J.M., Howard D.M. (1986). Changes in occurrence of Basidiomycete fruit bodies during forest stand development: With specific reference to mycorrhizal species. Trans. Br. Mycol. Soc..

[87] Wallander H. (2000). Uptake of P from apatite by Pinus sylvestris seedlings colonised by different ectomycorrhizal fungi. Plant Soil.

[88] Brundrett M. (1991). Mycorrhizas in natural ecosystems. Adv. Ecol. Res..

[89] Gibson F., Deacon J.W. (1988). Experimental study of establishment of ectomycorrhizas in different regions of birch root systems. Trans. Br. Mycol. Soc..

[90] Jones M.D., Durall D.M., Cairney J.W.G. (2003). Ectomycorrhizal fungal communities in young forest stands regenerating after clearcut logging. New Phytol..

[91] Giachini A.J., Oliveira V.L., Castellano M.A., Trappe J.M. (2000). Ectomycorrhizal fungi in Eucalyptus and Pinus plantations in southern Brazil. Mycologia.

[92] Chapela I.H., Osher L.J., Horton T.R., Henn M.R. (2001). Ectomycorrhizal fungi introduced with exotic pine plantations induce soil carbon depletion. Soil Biol. Biochem..

[93] Mead D.J. (2013). Sustainable Management of Pinus radiata Plantations.

[94] Kranabetter J.M., de Montigny L., Ross G. (2013). Effectiveness of green-tree retention in the conservation of ectomycorrhizal fungi. Fungal Ecol..

[95] Simard S.W., Beiler K.J., Bingham M.A., Deslippe J.R., Philip L.J., Teste F.P. (2012). Mycorrhizal networks: Mechanisms, ecology and modelling. Fungal Biol. Rev..

[96] Simard S.W. (2009). The foundational role of mycorrhizal networks in self-organization of interior Douglas-fir forests. For. Ecol. Manag..

[97] Crowther T.W., Stanton D.W.G., Thomas S.M., A’Bear A.D., Hiscox J., Jones T.H., Voříšková J., Baldrian P., Boddy L. (2013). Top-down control of soil fungal community composition by a globally distributed keystone consumer. Ecology.

[98] Koide R.T., Fernandez C., Malcolm G. (2014). Determining place and process: Functional traits of ectomycorrhizal fungi that affect both community structure and ecosystem function. New Phytol..

[99] Cozzolino V., Di Meo V., Monda H., Spaccini R., Piccolo A. (2016). The molecular characteristics of compost affect plant growth, arbuscular mycorrhizal fungi, and soil microbial community composition. Biol. Fertil. Soils.

[100] Boddy L., Hynes J., Bebber D.P., Fricker M.D. (2009). Saprotrophic cord systems: Dispersal mechanisms in space and time. Mycoscience.

[101] Zakaria A.J., Boddy L. (2002). Mycelial foraging by *Resinicium bicolor*: Interactive effects of resource quantity, quality and soil composition. FEMS Microbiol. Ecol..

[102] Harrington T.J. (2003). Relationships between macrofungi and vegetation in the burren. Biol. Environ..

[103] Eaton R.J., Barbercheck M., Buford M., Smith W. (2004). Effects of organic matter removal, soil compaction, and vegetation control on Collembolan populations. Pedobiologia.

[104] Wang Q.-K., Wang S.-L. (2008). Soil microbial properties and nutrients in pure and mixed Chinese fir plantations. J. For. Res..

[105] Crabtree C.D., Keller H.W., Ely J.S. (2010). Macrofungi associated with vegetation and soils at Ha Ha Tonka State Park, Missouri. Mycologia.

[106] Zheng Q., Hu Y., Zhang S., Noll L., Böckle T., Dietrich M., Herbold C.W., Eichorst S.A., Woebken D., Richter A. (2019). Soil multifunctionality is affected by the soil environment and by microbial community composition and diversity. Soil Biol. Biochem..

[107] He D., Xiang X., He J.-S., Wang C., Cao G., Adams J., Chu H. (2016). Composition of the soil fungal community is more sensitive to phosphorus than nitrogen addition in the alpine meadow on the Qinghai-Tibetan Plateau. Biol. Fertil. Soils.

[108] Shi L., Mortimer P., Ferry S.J.W., Zou X.-M., Xu J., Feng W.-T., Qiao L. (2014). Variation in forest soil fungal diversity along a latitudinal gradient. Fungal Divers..

